# A Review of Slipping Rib Syndrome: Diagnostic and Treatment Updates to a Rare and Challenging Problem

**DOI:** 10.3390/jcm12247671

**Published:** 2023-12-14

**Authors:** Isheeta Madeka, Sneha Alaparthi, Marisa Moreta, Shawn Peterson, Jeffrey J. Mojica, Johanes Roedl, Olubenga Okusanya

**Affiliations:** 1 Department of Thoracic Surgery, Thomas Jefferson University Hospitals, Philadelphia, PA 19107, USA; sneha.alaparthi@jefferson.edu; 2 Department of Physical Medicine and Rehabilitation, Thomas Jefferson University Hospital, Philadelphia, PA 19107, USA; marisa.moreta@jefferson.edu (M.M.); shawn.peterson@jefferson.edu (S.P.); 3 Department of Anesthesiology, Thomas Jefferson University Hospital, Philadelphia, PA 19107, USA; jeffrey.mojica@jefferson.edu; 4 Department of Radiology, Thomas Jefferson University Hospital, Philadelphia, PA 19107, USA; johanes.roedl@jefferson.edu

**Keywords:** slipping rib, slipping rib syndrome, chest pain, rib pain, costal cartilage, minimally invasive slipped rib repair, cartilaginous rib excision

## Abstract

Slipping rib syndrome (SRS) is a disorder that occurs when one or more of the eighth through tenth ribs become abnormally mobile. SRS is a poorly understood condition leading to a significant delay in diagnosis and therapeutic management. History and a physical exam are usually sufficient for a diagnosis of SRS. The utility of dynamic ultrasounds has also been studied as a useful diagnostic tool. Multiple surgical techniques for SRS have been described within the literature. Cartilage rib excision (CRE) has been the most common technique utilized. However, the literature has shown a high rate of recurrence and associated risks with the procedure. More recently, minimally invasive rib fixation and costal cartilage excision with vertical rib plating have been shown as successful and safe alternative techniques. This may be an effective, alternative approach to CRE in adult and pediatric populations with SRS.

## 1. Introduction

Slipping rib syndrome (SRS) is a disorder that occurs when one or more of the eighth through tenth ribs become abnormally mobile. It was first described by Cyriax in 1919; Davies Colley performed the first cartilaginous rib excision in 1922. Classical anatomic teachings state the eighth through tenth ribs have interchondral joints that fuse to form the costal margin and attach to the sternum. The mechanism of SRS is thought to be due to the loss of interchondral cartilaginous attachments, causing a defect in the costal margin. The tenth rib is the most affected rib. The subluxation of the lower rib cartilages manifests as sharp, radiating unilateral or bilateral chest wall pain caused by intercostal nerve irritation. The pain often radiates to the upper abdomen and/or back and is exacerbated by movement. Some patients report a clicking or popping sensation. Intercostal neuralgia is the most common symptom in patients with SRS. SRS is seen in children, adolescents, and adults with a predilection for females; the mean age at diagnosis is 19 years with most patients less than 40 years old. Its etiology is multifocal, including trauma, hypermobile connective tissue disorder, and/or degenerative changes. Trauma likely remains the most common etiology, both direct (i.e., major injury or blow disrupting muscles and ligaments) and indirect (i.e., with extreme respiratory movements, rapid movements). Indirect trauma is more common and is more difficult to isolate for patients and providers. SRS is a poorly understood condition leading to a significant delay in diagnosis and therapeutic management [1]. Due to under-diagnosis, there are no accurate data regarding its prevalence [2].

Lawsi et al. aimed to determine the incidence of slipped rib syndrome by evaluating forty cadavers for the mobility and attachment of the ninth and tenth ribs. The authors found 100% of the cadavers’ ninth rib was attached to the eighth rib by an interchondral cartilaginous attachment. A total of 33% of cadavers had ninth ribs with stable costal margin and no mobility, 37% had moderate mobility, and 30% had high mobility. In addition, 19% had ninth rib subluxation, with 100% with subluxation into the chest. In comparison, only 18% of cadavers’ tenth rib was attached to the ninth rib and 4% of the tenth ribs had no mobility, whereas 86% had high mobility. In total, 59% had a “floating” tenth rib, with 33% having subluxation. Of the tenth ribs, 10% had a “hooked” rib tip, often seen intra-operatively in patients with SRS; there was a significant association with patients with a “hooked” tip and internal subluxation. There was no significant difference in patients with tenth rib subluxation across sex, age, heigh, weight, or laterality. The study showed hypermobility in the ninth and tenth ribs is common and may predispose patients to SRS. It also emphasized the importance of careful intra-operative evaluation prior to surgical intervention, as it is possible to misinterpret the tenth versus eleventh rib given the tenth rib was more commonly “floating”. This finding is most often classically taught with regards to the eleventh and twelfth rib. Thus, it would be possible to misidentify the ribs and perform an ineffective operation. The study is limited due to the age of the cadavers (mean 83 years) which is discordant with the age of diagnosis of SRS patients. The past medical and surgical history of the cadavers was unknown; as such, any incidence of indirect or direct trauma is unknown [3].

Current conservative treatment modalities include activity modifications, topical and oral analgesia with NSAIDs and/or opioids, osteopathic manipulation (OMT), and intercostal nerve blocks [3]. Multiple surgical techniques for SRS have been described within the literature as well. Traditionally, costal cartilage excision has been the most common technique utilized. However, minimally invasive rib fixation and costal cartilage excision with vertical rib plating have been described in more recent years [1,4,5,6].

## 2. Etiology

**Figure 1:**
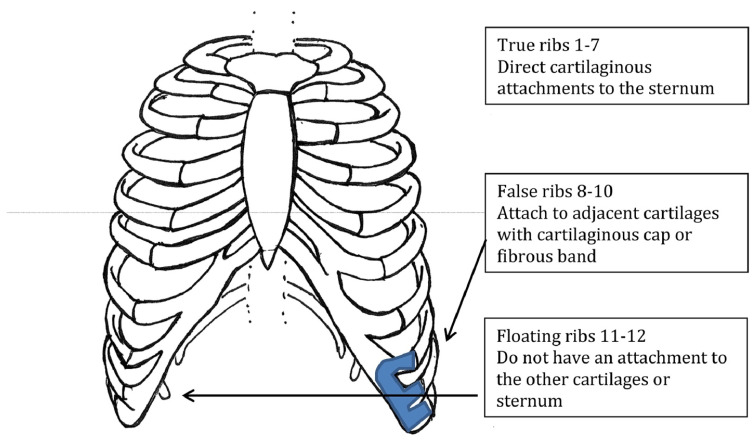
Normal rib anatomy with differentiation of rib classifications [7].

Traditional anatomy teachings classify ribs into true ribs (ribs 1–7), false ribs (ribs 8–10), and floating ribs (ribs 11–12) (Figure 1). True ribs have direct cartilaginous attachments to the sternum. False ribs attach to adjacent cartilages with a cartilaginous tip or fibrous band. Floating ribs do not have an attaching to the other cartilages or sternum. Given the false ribs or ribs 8–10 not having direct cartilaginous attachments to the sternum, they are most often implicated in SRS [7].

**Figure 2:**
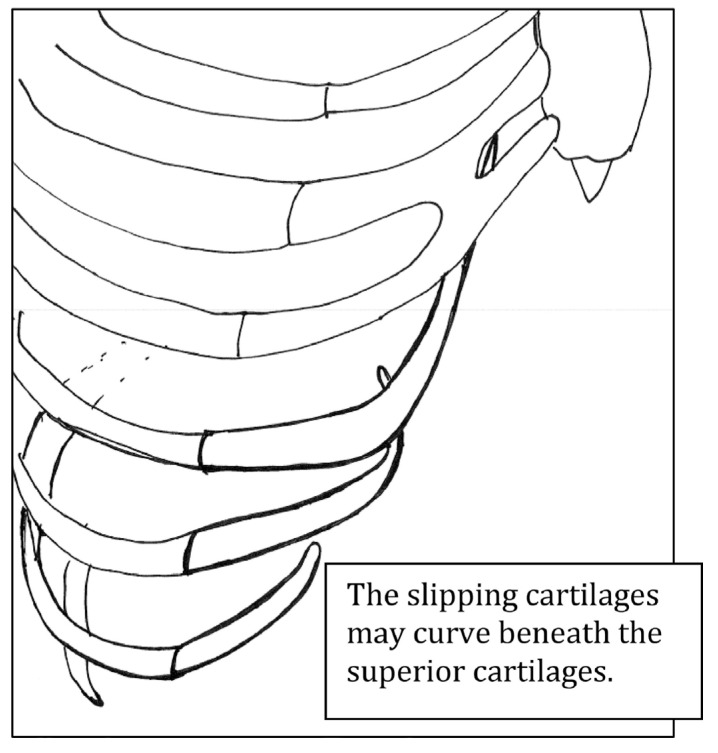
Pathophysiology of slipped rib syndrome [7].

A defect of the costochondral cartilage is the primary mechanism of SRS, most often seen within the interchondral ligaments of anterior ribs 8th through 10th. Hypermobility (associated with or without connective tissue disorders such as Marfan’s syndrome and Ehlers-Danlos syndrome), disruption of the fibrous articulation, or congenital/developmental deformities can all cause weakness in the costochondral cartilage. The weakness subsequently causes laxity of the rib and “slips” above and below the adjacent superior rib (Figure 2). Hypermobility may have an association with hormones that increase joint laxity. Female athletes (specifically swimmers, gymnasts, etc.) are at a greater risk of developing SRS due to repetitive movement patterns, and joint laxity that may be affected by hormones [8].

In 1975, McBeath and Keane examined 20 specimens of cartilages from 8th, 9th, and 10th rib articulations from ten autopsy cases. In all specimens, “slipping” or subluxation of the rib tip above or below the adjacent superior rib was only possible when the fibrous tissue between them was incised [9]. This further supports the theory that trauma, direct or indirect, is likely an important cause of SRS.

## 3. Challenges

There is a lack of familiarity of SRS among medical providers. Furthermore, its clinical symptomology often mirrors other thoracic and abdominal pathology, such as costochondritis, intercostal muscle strain, or gallbladder disease. Patients often see multiple providers and undergo a multitude of diagnostic testing and/or invasive procedures [10]. In an adult cohort of patients with SRS examined by Hansen et. al, patients were seen by multiple specialists (median, two physicians) and underwent several diagnostic imaging studies (median, two studies). More specifically, 40% of patients underwent at least one invasive test or non-therapeutic surgical procedure. In total, 19% of patients underwent laparoscopic cholecystectomy for pain with no relief [1]. These data have also been corroborated within the pediatric population [3,8]. In Lawsi et al., 50% of pediatric SRS patients had a previous diagnosis for their pain, with the most common being costochondritis (21%) or biliary disease (36%). In a pediatric SRS cohort examined by Gould et al., patients underwent a median number of two pre-diagnosis imaging studies. Diagnostic evaluation also included colonoscopy and esophagogastroduodenoscopy in 14% and 10% of patients, respectively. There were 12% of pediatric patients under laparoscopic cholecystectomy without symptom relief and 93% of pediatric SRS patients were seen by a specialist other than the surgeon; the most commonly referred specialties included gastroenterology, pain management, and physical therapy [4]. In a 2022 single-institution study by Macgregor et al., a pediatric patient underwent fourteen invasive and non-invasive tests and was given an intractable chronic pain diagnosis [10]. The lack of familiarity with the pathology leads to a significant delay in diagnosis and treatment, as well as excessive diagnostic testing, without any substantial symptom relief. The median time from symptom onset to surgical repair is reported as high as 18 months in the adult population and 2.5 years in the pediatric [1,10]. In McMahon et al., the mean age at symptom onset was 15.8 years, versus the mean age at diagnosis which was 17.7 years, further illustrating the delay in diagnosis [6].

This delay in diagnosis and treatment has a profound effect on patients’ mental health. One third of patients in an adult SRS cohort had suicidal ideations due to pain; three patients had attempted suicide [1]. Other studies have shown an increased likelihood of SRS patients linked with a psychiatric diagnosis, including depression. This may be linked with ongoing chronic pain, as well as the overall strain between the patient and provider given the delay in diagnosis of slipped rib syndrome in these patients.

## 4. Diagnostics

**Figure 3:**
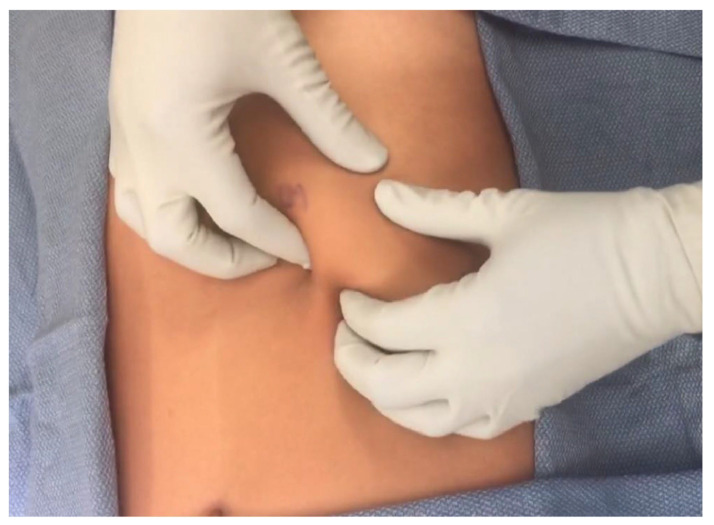
“Hooking” maneuver [7].

History and physical examination are usually sufficient for a diagnosis of SRS. In 1977, Heinz and Zavala describe the “hooking” technique, which involves hooking the fingers under the costal margin and pulling the rib superiorly and anteriorly (Figure 3) [11].

This reproduces subluxation and intercostal nerve impingement and elicits pain in patients with SRS [6]. However, this technique is painful. In a 2016 study, Gould et al. found limited utility in the hooking maneuver. Instead, they utilized gentle palpation of the costal cartilage at the point of maximum tenderness at the anterior costal cartilage junctions. In a 2018 review, McMahon shares another physical exam maneuver in which palpation along the lower cartilages may help identify disconnected cartilages associated with movement; following the costal margin contour, the examiner may be able to palpate and elicit point tenderness where the cartilage curls beneath the overlying rib cage. This is a less painful maneuver than the hooking technique [7]. In a 2020 study, Hansen et al. proposes the following physical exam criteria to diagnose SRS: (1) a palpable separation of at least 1 cm at the anterior insertion of the 10th rib into the costal arch, (2) the 10th rib is unusually mobile on palpation, and (3) palpation at the separation point reproduces the patient’s pain.

Computed tomography, magnetic resonance imaging, or bone scintigraphy are often not helpful in diagnosis as they do not show costal cartilage separation. However, imaging may be helpful in delineating the aberrant rib anatomy and thus, help with surgical planning. It may also be helpful in excluding other causes, both thoracic and abdominal [1].

Hansen et al. showed that clinical findings of SRS correlated with operative findings in 100% of patients, underscoring the importance of history and physical examination as an important part of diagnosis. In contrast, the authors found that while 76% of the cohort with SRS had prior imaging studies, none confirmed SRS [1]. In a pediatric SRS cohort examined by Fraser et. al, imaging did not show any diagnostic value; all diagnoses were confirmed via a physical exam or upon surgical resection [4]. However, neither study cohort utilized dynamic ultrasound for diagnosis.

The use of ultrasound has been shown to aid in the diagnosis of SRS. In a prospective analysis by Romano et al., four patients underwent surgical correction of SRS; a pre-operative ultrasound was used to measure the thickness of the rectus abdominus muscle, which is innervated by the intercostal nerves seventh through eleventh. The study found that there was a 0.25 cm decreased thickness of the rectus abdominus muscle at the insertion of the xiphoid process in patients with SRS when compared to healthy controls. Romano et al. postulated this difference as being due to chronic intercostal nerve entrapment, and therefore, subsequent hypotrophy of the rectus abdominus muscle, which may lead to additional instability in patients with SRS [12].

More specifically, dynamic ultrasound imaging of the ribs has also been shown as a useful diagnostic tool and with an experienced sonographer, it can be a valuable tool for differentiating causes of costal pain. In a 2019 retrospective review by Van Tassel et al., dynamic ultrasound imaging accurately diagnosed SRS in 89% of cases when compared with clinical examination using the hooking maneuver or surgical confirmation of slipping rib. Dynamic maneuvers, specifically the active crunch and passive rib push maneuvers, were utilized. During the crunch maneuver, the patient was instructed to raise their head to contract the abdominal muscles; the rib push maneuver involved placing a finger below the rib tip of interest. Patients with SRS will show the medial head of the costal cartilage of the slipped rib moving superficially or deep to the adjacent rib. An ultrasound is able to detect cartilaginous fusion immediately above the symptomatic level as well as increased intercostal soft tissue echogenicity at the symptomatic level, both likely sequelae of inflammation. These imaging findings coupled with the mobility of ribs seen on an ultrasound during crunch or push maneuvers were diagnostic of symptomatic SRS. Ultrasounds also confirmed SRS’s absence in 100% of cases [2]. SRS may be an asymptomatic diagnosis. Therefore, reproducing the pain with a dynamic ultrasound is what confirms the diagnosis.

## 5. Treatment

### 5.1. Non-Surgical Treatment

The conservative management of SRS begins with the control of inflammation and pain. Decreased activity that exacerbates symptoms, oral NSAIDs, and ice are often first-line non-surgical therapies. The use of heat, physical therapy, and/or topical NSAIDs have also been used for symptom management. However, conservative management pathways may often prolong the course prior to a definitive cure with surgical intervention; thus, Gould et al. recommends surgical excision upon a diagnosis of SRS [13].

Local anesthetic intercostal nerve blocks and/or steroid injections may be used as a diagnostic and therapeutic modality. They have been shown to offer temporary relief in patients with SRS. It has been shown to provide complete symptomatic relief in some SRS patients. This may be an important option to consider in patients who want to avoid surgery. A local nerve block may also help with pre-operative planning by identifying the specific slipped rib that should be surgically corrected [7]. There has been a documented case report of a botulinum toxin injection with a shorter-term pain relief compared to a local nerve block [14].

### 5.2. Surgical Treatment

**Figure 4:**
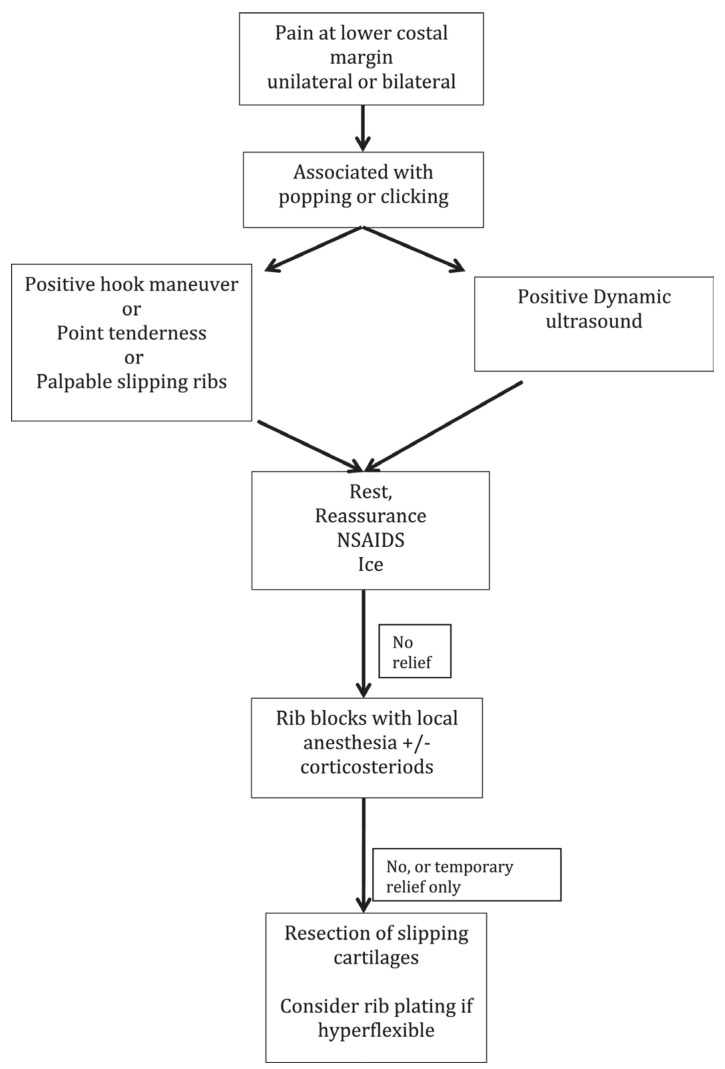
Algorithm for diagnosis and treatment for SRS [7].

If conservative treatment fails, surgical excision is recommended based on established algorithms by McMahon (Figure 4). Cartilaginous rib excision (CRE) has been the mainstay of surgical treatment for SRS. This usually involves the resection of two or three rib cartilages. However, in recent years, minimally invasive rib stabilization and cartilaginous rib excision with vertical rib plating has been reported.

CRE is performed via a small incision at the lower costal margin; the hypermobile cartilaginous rib is identified and excised to the costochondral junction. The rib is left intact, the thoracic cavity is not violated, and the neurovascular bundle is carefully preserved. The current literature shows symptomatic relief following CRE in adult and pediatric SRS patients. In a 2022 single-institution descriptive study, Macgregor et al. reviewed thirteen pediatric patients with SRS who underwent CRE from 2012 to 2020. In total, 91% of patients reported improvement in pain post-operatively, with a median follow-up time of 3.5 months. Unfortunately, this cohort had a median time of 2 years from symptom onset to surgical resection, which is supported by the literature [10]. Fraser et al. shows similarly high rates of symptom resolution (77%) in pediatric patients undergoing cartilaginous rib excision over a 4.5-year follow-up period. This study also showed a high long-term satisfaction rate over a long follow-up period among patients who underwent CRE, even if a complete cure was not reached [4]. In a retrospective study by Mazzella et al., nineteen adult patients with SRS showed that CRE led to a resolution of symptoms in the early post-operative period; however, the mid- to long-term follow-up showed symptom recurrence in 31% of patients. The main challenge of utilizing CRE is that it does not resolve the rib hypermobility, it only decreases how much rib is available to cause irritation [15].

Laparoscopic CRE has also been studied. In 2020, Squillaro et al. performed a small, retrospective review of four pediatric patients with SRS comparing laparoscopic versus open CRE. The study showed laparoscopic CRE is a safe and effective technique. Three 5 mm ports are placed at the umbilicus, mid-epigastric, and mid-suprapubic. A laparoscopic hook cautery is used to open the peritoneal lining, dissect, and expose the cartilage; a Maryland or locking grasper helps provide traction. A pituitary rongeur is used to cut the cartilage and is removed via the umbilical port site. The peritoneal lining is laparoscopically closed. The average procedure time for laparoscopic CRE was 73 min; this was longer than the 57 min procedure time for open CRE. Within the laparoscopic CRE population, the average length of the cartilage resected was 2.3 cm with 1.3 cartilages resected. The length of stay was similar between both groups; 1.3 days with laparoscopic CRE versus 1 day with open CRE [16].

Likely due to the continued rib hypermobility, there are high rates of recurrence in patients with SRS who undergo CRE. Gould et al. reports nearly a fourth of patients will experience recurrence of SRS after CRE, citing 26% requiring subsequent reoperation [13]. Within the pediatric cohort in Fraser et al., 22% of patients had recurrence following CRE requiring reoperation with a median time to recurrence of 1.6 years. No anthropometric differences were found between patients who did and did not have recurrence requiring reoperation. However, patients with recurrence requiring reoperation were found to have residual cartilage and/or hypermobile ribs that slipped intra-operatively; Fraser et al. postulate this may be due to regrowth from repeated trauma in the pediatric SRS population [4]. There are no long-term studies defining recurrence rates and risk factors in the adult SRS population.

Vertical rib plating has been shown to decrease recurrence rates. In a 2020 retrospective review of 85 patients by McMahon et. al, two cohorts of SRS patients who underwent cartilaginous rib excision were compared, those with and those without bioabsorbable vertical rib plating (VRP). Rib plating was utilized to reduce recurrence and reoperation if the surgeon was able to manually subluxate the affected rib over, under or into the adjacent rib during intra-operative evaluation. The BioBridge^TM^ bioabsorbable plate was utilized. Bioabsorbable plates have been used for other pathologies, such as facial fracture repair and clavicle fixation. They have an improved profile when compared to metal implants and have been shown to be safe in adult and pediatric populations. Over time, the plates are absorbed and leave behind scar tissue that serves as a stabilization for the ribs. A total of 17.1% of patients who were not plated experienced recurrence, versus 3.4% for patients who were plated, a significant reduction. Notably, in the plated patient cohort who presented with recurrence, both patients presented with clinical symptoms after a motor vehicle crash and a pedestrian struck by a motor vehicle crash. Of the 7 patients who were unplated and recurred, 6 patients underwent more than one reoperation to remove additional symptomatic rib or cartilage. Of the 41 patients within the cohort, three patients were unplated, recurred, and subsequently plated. Recurrence was defined as the return of clinical symptoms and confirmed with physical exam findings and positive dynamic ultrasounds. The median time to recurrence was six months. VRP significantly increased the intra-operative time (48 min in bilateral VRP, *p* < 0.001) and length of stay (11 h, *p* < 0.001). VRP did not increase the frequency of severe post-operative pain events. There were no major complications associated with VRP. Whilst vertical rib plating appears promising in the short-term, long-term data are still necessary to evaluate recurrence rates after the bioabsorbable plates have completely resorbed, which occurs by the second year of use. Within this study, the decision to utilize vertical rib plating was dependent on intra-operative subluxation of the affected ribs; further research is also needed to determine what criteria should be used when selecting patients who should or should not undergo VRP [6].

Chest wall reconstruction is a challenging undertaking. The ideal reconstructive material restores skeletal integrity, is low risk, easy to use, and inexpensive. Reconstruction materials include synthetic and biomaterials. Synthetic meshes include polypropylene, polytetrafluoroethylene (PTFE), polyglactin, and polypropylene mesh-methyl methacrylate composite. However, they did not completely incorporate into tissue and are susceptible to infection; they require removal if infected. Biomaterials have more recently been introduced in thoracic skeleton reconstruction. In a retrospective review of 112 patients who underwent chest wall stabilization or reconstruction, 25 patients (22%) underwent reconstruction with biologic materials, a bovine pericardium patch and/or polylactic acid (PLA) bars (BioBridge™, Acute Innovations, Hillsboro, OR). In total, 68% of patients underwent chest wall resection and reconstruction for malignant disease, 44% underwent thoracic radiation pre-operatively, and 40% had pre-operatively infected reconstruction sites. Even within this population with a multitude of high-risk factors for chest wall reconstruction failure, only three patients required the removal of their biomaterials. In two patients, bovine pericardium was explanted during reoperation for partially necrotic muscle. In one patient, PLA bars were explanted for inflammatory reaction 3 months after a redo pectus repair. The median follow-up was 12 months; no patients with pre-operatively infected sites required biomaterial removal. No patients had a paradoxical respiratory motion of the thoracic skeleton, seroma formation, wound infection, or biomaterial infection [17]. 

However, there have been reports of the failure of bioabsorbable plates used for chest wall reconstruction. Haslem et al. illustrates the case of a 66-year-old woman who underwent chest wall reconstruction for a radiation-induced wound with exposed ribs. Surgical resection and reconstruction included the excision of two ribs with a bony defect measuring 10 cm by 10 cm. Two BioBridge^TM^ bioabsorbable plates were used along with a biologic Strat Tice porcine tissue matrix as part of the reconstruction to provide chest wall stability and good cometic outcome. The soft tissue defect was covered with a myocutaneous latissimus dorsi flap. Six months after the index operation, the patient presented with a 2.5 cm superficial wound dehiscence and was found to have a 2 cm fragment of BioBridge™ plate which was extracted. On reoperation, the two BioBridge™ plates were fragmented in seven pieces, which were explanted. There was no plate resorption at six months. The authors postulated the structural failure of the BioBridge™ plates were likely contributory in the patient’s post-operative wound complication [18]. BioBridge states the plates maintain their strength and durability for up to 6 months and resorption via hydrolysis occurs between 18 and 24 months.

In a 2019 retrospective review, Hansen et al. describes a minimally invasive slipped rib repair in 29 adult patients with SRS without cartilaginous rib excision. The authors stabilize the tenth rib costal insertion with two figure-of-eight stitches using an orthopedic tape suture (TigerTape) by fixing it superiorly and inferiorly. The sutures are carefully placed so as to avoid nerve entrapment. The incision is closed in multiple layers. The procedure is completed without entering the pleural cavity, so no thoracostomy tube is necessary. Patients were discharged the same day with an oral pain regimen. On post-operative follow-up, patients had a significant increase in function after slipped rib repair at one week, one month, and six months post-operatively (*p* < 0.001). They also had a significant decrease in disability during the same time intervals (*p* < 0.001) [1].

The reports of combining CRE and VRP with using the patients’ own excised cartilage (from CRE) as spacers between the ribs are forthcoming. Utilizing CRE as spacers may help further stabilize the region and prevent nerve impingement if suture fixation of the affected ribs is performed. There may be utility in extending this rib fixation technique to the pediatric population; however, there may be challenges applying the procedure to growing children, in whom the rib cage is still undergoing somatic growth.

Pre-operative, intra-operative, and post-operative pain control in patients who undergo minimally invasive slipped rib repair is not well-understood. There is a paucity of literature studying pain management in this population. Macgregor et al. underscores the need for a multidisciplinary approach. There is also benefit in the implementation of a formal pathway that includes multiple specialties, including regional and pain anesthesiologists, physical therapy, and pain psychologists. This would allow patients to undergo a coordinated, monitored, and tailored non-operative management plan for symptom relief. This may be useful in selecting patients who are failing non-operative management and would benefit from surgical intervention [1].

Most patients are taking either neural modulating medications, NSAIDs, or narcotics pre-operatively; many on a multi-modal analgesic regimen. Intercostal nerve blocks are utilized intra-operatively. Post-operatively, patients can be discharged the same day with a multi-modal pain regimen, often including narcotic medications and NSAIDs. In Hansen et al.’s cohort of 29 patients, a similar intra-operative and post-operative regimen was utilized. Median post-operative improvement in pain at 1 month and 6 months were 75% (*p* < 0.001) and 80% (*p* < 0.001). Among the patients who used pain medications pre-operatively, 100% of patients discontinued narcotics at 1 month, 86% discontinued neural modulators and 92% discontinued NSAIDs (all values *p* < 0.001) [1]. Burjek et al. reports on the successful use of an ambulatory erector spinae plane (ESP) block in a seventeen-year-old patient with a history of chronic pain and SRS after surgical intervention. The patient underwent intra-operative ESP placement at level T9 through T10. Post-operatively, she received a continuous nerve catheter infusion of 10.8 mL/h of ropivacaine 0.2% (0.125 mL/kg/h), as well as gabapentin 600 mg three times a day, ketorolac 30 mg every six hours, acetaminophen 650 mg every 6 h, diazepam 2.6 mg every 6 h as needed, and demand-only hydromorphone patient-controlled analgesia (PCA). She was discharged home on post-operative day one with an elastomeric pump and an oral regimen of gabapentin 600 mg three times a day, acetaminophen 650 mg every 6 h, ibuprofen 600 mg every 6 h, cyclobenzaprine 10 mg as needed, and oxycodone 5 mg as needed. The ESP catheter was removed at home on post-operative day four [19]. Large studies have shown the use of peripheral nerve catheters in children is safe, with low failure and complication rates [20]. In a randomized control trial, Gould et al. compared ESP and continuous thoracic epidural analgesia in adult cardiac surgery patients who underwent sternotomy and showed comparable outcomes, suggesting ESP is a safe and effective alternative [21]. However, the use of ambulatory ESPs for acute post-operative surgical pain has not been studied in adult patients.

Intercostal nerve cryoablation has been used in the minimally invasive repair of pectus excavatum. Cryoablation (or cryo) involves the thoracoscopic (either intra- or extra-thoracically) delivery of extremely cold temperatures to temporarily stunt nerve function via a probe. This loss of function does not seem to have an effect on the motor function of breathing due to its locoregional delivery. A study of pediatric SRS patients by Lai et al. compared those who underwent CRE without cryo, those who underwent CRE with cryo, and those who underwent CRE with minimally invasive pectus excavatum repair and cryo. Of note, CRE was performed with vertical rib plating with a BioBridge™ absorbable plate. Within the CRE with cryo cohort of 20 patients, cryo was delivered extra-thoracically by tunnelling the problem below the intercostal muscle to provide temporary disruption of the intercostal nerve. The study reported there was a significant decrease of in-hospital opioid use and length of stay in patients who underwent cartilaginous rib excision and vertical rib plating with cryoablation (median LOS 1 day) versus cartilaginous rib excision without cryoablation (median LOS 2 days). Alternatively, the number of CREs performed, unilateral versus bilateral CRE, use of rib plating, age, sex, or BMI were not associated with in-hospital use. These data are corroborated by studies within the minimally invasive repair of the pectus excavatum population, which show decreased opioid use and length of stay in patients who undergo cryoablation versus those who do not [5,22]. No patients had post-operative abdominal wall laxity at a two-week follow-up. This is an important finding as the manufacturer warns against using cryoablation below the ninth rib as those nerves are contributory in motor innervation of the abdominal wall muscle [23]. There is still further research to be conducted on the long-term utility of cryoablation in patients undergoing slipped rib repair and its long-term effects on post-operative pain control and possible complications, such as abdominal wall weakness or pneumothorax.

## 6. Conclusions

SRS is a poorly understood diagnosis that affects both adults and children leading to a significant delay in diagnosis and unnecessary diagnostic tests. Early and accurate diagnosis coupled with timely referral to a surgical provider with expertise in SRS is the most important aspect of treatment. Surgical techniques to treat SRS are continuously evolving to improve long-term, post-operative pain relief and recurrence. Currently, SRS recurrence is not well-delineated; further research is necessary to determine the risk factors that may increase the chance of recurrence. The minimally invasive slipped rib repair described by Hansen et al. may be an effective, alternative approach to CRE in adult populations with SRS. However, the utility of CRE and vertical rib plating by McMahon et al. has also shown promising results. There have been no cases that have combined Hansen’s approach of minimally invasive rib stabilization with suture fixation with the use of bioabsorbable rib plating in either adult or pediatric populations. There have also been no cases that have combined the CRE and vertical rib plating in McMahon et al. with the use of excised rib cartilage as spacers, and with suture fixation for further stabilization. There have been no randomized control trials comparing the post-operative outcomes and recurrence in patients who undergo the above-mentioned techniques for repair. However, the long-term utility of bioabsorbable rib plating and possible post-operative complications has also not been studied. Moreover, pain management utilizing cryoablation, radiofrequency ablation, peripheral nerve catheters, and multimodal oral pain regimens is an important facet of pre-, intra-, and post-operative care in this population. While there have been established pathways to help guide non-operative management, further research is necessary to identify which patients would benefit from which surgical intervention. There have been promising diagnostic and therapeutic updates regarding the management of SRS. However, there remain large gaps in the literature that deserve further study.
